# Inter-Specific and Intra-Specific Competition of Two Sympatrically Breeding Seabirds, Chinstrap and Gentoo Penguins, at Two Neighboring Colonies

**DOI:** 10.3390/ani11020482

**Published:** 2021-02-11

**Authors:** Won Young Lee, Seongseop Park, Kil Won Kim, Jeong-Hoon Kim, Jong-Ku Gal, Hosung Chung

**Affiliations:** 1 Division of Polar Life Sciences, Korea Polar Research Institute, Incheon 21990, Korea; sspark2730@gmail.com (S.P.); jhkim94@kopri.re.kr (J.-H.K.); hchung@kopri.re.kr (H.C.); 2 Division of Life Sciences, Incheon National University, Incheon 22012, Korea; kilwon@inu.ac.kr; 3 Division of Polar Ocean Sciences, Korea Polar Research Institute, Incheon 21990, Korea; jkgal@kopri.re.kr

**Keywords:** inter-specific competition, intra-specific competition, niche partitioning, Chinstrap penguin, Gentoo penguin

## Abstract

**Simple Summary:**

Under limited resources, different species may coexist by segregating their food items and foraging time, but neighboring colonies within the same species may have highly overlapping foraging habits. Thus, it is hypothesized that intra-specific competition is more intense than the inter-specific competition. To test this hypothesis, we chose sympatrically breeding Chinstrap and Gentoo penguins at two neighboring colonies and tracked their foraging space and dive depths during chick-rearing. Here we report that there was a larger overlap in the foraging space between the two species than within each species, whereas there was lower inter-specific isotopic niche overlap than intra-specific isotopic niche overlap. Despite the low intra-specific spatial overlap, diets of conspecifics remained similar, resulting in the higher isotopic niche overlaps. Our results suggest that intra-specific competition is higher than inter-specific competition due to a lack of niche partitioning.

**Abstract:**

Theory predicts that sympatric predators compete for food under conditions of limited resources. Competition would occur even within the same species, between neighboring populations, because of overlapping foraging habits. Thus, neighboring populations of the same species are hypothesized to face strong competition. To test the hypothesis that intra-specific competition is more intense than inter-specific competition owing to a lack of niche partitioning, we estimated the foraging area and diving depths of two colonial seabird species at two neighboring colonies. Using GPS and time-depth recorders, we tracked foraging space use of sympatric breeding Chinstrap and Gentoo penguins at Ardley Island (AI) and Narębski Point (NP) at King George Island, Antarctica. GPS tracks showed that there was a larger overlap in the foraging areas between the two species than within each species. In dive parameters, Gentoo penguins performed deeper and longer dives than Chinstrap penguins at the same colonies. At the colony level, Gentoo penguins from NP undertook deeper and longer dives than those at AI, whereas Chinstrap penguins did not show such intra-specific differences in dives. Stable isotope analysis of δ^13^C and δ^15^N isotopes in blood demonstrated both inter- and intra-specific differences. Both species of penguin at AI exhibited higher δ^13^C and δ^15^N values than those at NP, and in both locations, Gentoo penguins had higher δ^13^C and lower δ^15^N values than Chinstrap penguins. Isotopic niches showed that there were lower inter-specific overlaps than intra-specific overlaps. This suggests that, despite the low intra-specific spatial overlap, diets of conspecifics from different colonies remained more similar, resulting in the higher isotopic niche overlaps. Collectively, our results support the hypothesis that intra-specific competition is higher than inter-specific competition, leading to spatial segregation of the neighboring populations of the same species.

## 1. Introduction

Theory predicts that predators with common prey items occupy overlapping ecological niches, and competitions may arise for the limited resources [1,2,3]. This may result in resource partitioning, leading to more distinctive niches among the predators [4,5,6,7]. For instance, competing species may differ their foraging times in the day or at night [8,9,10] or preferentially consume different sizes of the same prey [11,12,13].

Competitions occur not only between different species, but also within the same species, among neighboring populations. Intra-specific competition is intense due to the lack of niche partitioning [14,15,16], and may lead to segregation of foraging areas [17]. Previous studies have tracked the foraging habitats of neighboring populations of the same species and estimated their competition [17,18,19].

Many seabirds are colonial breeders, with mixed-species populations occupying the same habitat [18,20,21], and several breeding colonies with favored nesting places are often closely located. During the breeding season, parent seabirds are restricted to foraging areas near the colonies, needing to be able to commute to and from such foraging areas, making them “central place foragers” [15,22,23]. Thus, seabirds with similar breeding periods and/or restricted foraging habitats often face competition due to the limited availability of resources within a niche, which leads to segregation of areas between the species. Such a segregation of foraging areas suggests that inter-colony overlaps would occur in areas where birds aggregate for highly productive food sources, often located remotely from multiple colonies [24]. Thus, seabirds are a good model taxon to estimate and compare the extent of both inter- and intra-specific competitions.

Penguins are diving seabirds, and one of the most extensively studied central place foragers [25,26,27]. In the Antarctic Peninsula and the sub-Antarctic islands, Chinstrap (*Pygoscelis antarcticus*) and Gentoo penguins (*Pygoscelis papua*) may often breed in shared colonies. Gentoo penguins have been reported to be flexible, consuming fish, cephalopods, and amphipods, whereas Chinstrap penguins are krill-specialist predators [18,28]. During chick-rearing, however, both species mainly feed on the same main prey item, the Antarctic krill (*Euphausia superba*) in the South Shetland Islands [12,29]. The two penguins differentiate their ecological niches by their time of foraging, i.e., both species can avoid overlaps in foraging hours [30], or in their use of the foraging space, i.e., the former feed in off-shelf areas, whereas the latter prefer the on-shelf areas [12,30,31]. Previous studies have focused mainly on inter-specific competition between species or intra-specific competition between neighboring colonies, and the simultaneous comparison of inter- and intra-specific competition has received little attention.

To estimate the intra- and inter-specific competition regarding foraging areas, we investigated the space-use behavior of Chinstrap and Gentoo penguins from two neighboring colonies, i.e., Ardley Island and Narębski Point, during the chick-rearing period. Using GPS and depth loggers, we visualized the utilization of the foraging area by these penguins and estimated their vertical dive depth in the same foraging area on the same days. We further employed blood stable isotope analysis to determine the differences in the foraging locations and prey trophic levels between the species and populations. Stable isotope ratios of carbon (δ^13^C) were used to determine the dominant feeding area, i.e., onshore or pelagic, and nitrogen isotope ratios (δ^15^N) were used to estimate the prey trophic levels [32,33,34]. The main objectives of the study were to determine (1) the intra- and inter-specific segregation of the foraging space between the two species from the two neighboring colonies, and (2) differences in prey consumption based on stable isotope values. Based on the intra- and inter-specific competition hypothesis, we predicted that intra-specific competition is more intense than inter-specific competition. Accordingly, the degree of intra-specific foraging niche segregation was expected to be higher than that of inter-specific segregation.

## 2. Materials and Methods

### 2.1. Study Populations and Field Survey

Chinstrap and Gentoo penguin colonies were surveyed on Ardley Island (hereafter AI; 62°13′ S, 58°54′ W; Antarctic Specially Protected Area No. 150) and Narębski Point (hereafter NP; 62°14′ S, 58°46′ W; Antarctic Specially Protected Area No. 171). The two colonies are located across the bay, approximately 8 km apart. Both study species are sympatric breeders; hence, the two colonies were ideal for simultaneous inter- and intra-specific comparisons. The breeding numbers varied between the colonies. At AI, few Chinstrap pairs and 4000–5000 Gentoo pairs are recorded annually [35]. In the 2017–2018 season, 20 Chinstrap and 7227 Gentoo breeding pairs were documented at AI [36], whereas 2918 Chinstrap and 2604 Gentoo breeding pairs were recorded at NP in the same season [35]. Subsampling from 500 nests showed that the Gentoo penguin chicks hatched on 17 December, whereas the Chinstrap penguin chicks hatched on 23 December [32,33] (hatching dates indicate the mean values from the subsampled nests).

GPS and depth recorders were deployed on randomly selected adult breeding Gentoo penguins (*n* = 20 at NP, 12 at AI) on 27 December 2017, and on Chinstrap penguins (*n* = 19 at NP, 13 at AI) on 3 January 2018. Birds heading to the coast to leave on a foraging trip were captured using a net. We had observed the breeding pairs with two chicks at their nests. When one of the parents had headed to the coast after the breeding shift, we followed the individual. All captured birds were rearing two 7–14-day-old chicks. We used a GPS-depth dual recorder (GPL400-D3GT, Little Leonardo Corporation, 20 mm diameter, 103 mm long, weighing 57 g; at 1 s sampling rate for location and depth; *n* = 8) and a combination of separate GPS (F3G-133A, Sirtrack, length 63 mm, width 24 mm, height 22 mm, mass 31 g, sampling rate 30 s) and time-depth recorders (M190-DT, Little Leonardo Corporation, diameter 12 mm, length 45 mm, mass 5 g, shape cylindrical, sampling rate 1 s; ORI400-D3GT, diameter 12 mm, length 45 mm, mass 9 g, sampling rate 1 s). GPS and depth recorder combinations are provided in Supplementary Materials Table S1. All devices were attached to the dorsal feathers using Tesa tape as adhesives and then Loctite glue was supplemented on the surface of the tape to ensure attachment. In all birds, it took less than 15 min for deployment.

All devices weighed less than 1.5% of the total weight of the penguins, and no abnormal behaviors were observed during attachment. All 64 penguins returned to their nests within 1–2 days after deployment. Near the nest sites, all penguins were recaptured, and the devices were successfully retrieved. The recapture procedure took less than 5 min. Three penguins did not have any dive records (one Chinstrap at NP, and one Gentoo and one Chinstrap at AI). Of the 40 penguins with successful round-trip records, eight individuals undertook two foraging trips (two Gentoo and four Chinstrap penguins at NP, and one Gentoo penguin at AI). Thus, a total of 47 foraging trips with GPS location and depth data were available for analyses. The detailed information on the numbers of GPS and depth recorders were provided in Supplementary Materials Table S1.

### 2.2. GPS and Depth Data Analyses

All spatial analyses were performed in ArcMap^®^ (version 10.8, ESRI, Redlands, CA, USA). Diving indices were analyzed using Igor Pro software (version 6.32, WaveMetrics, OR, USA). We assumed that the points exceeding a maximum velocity of 12 km/h per point were not realistic movements (error points) [37]. We filtered such points using Movement Ecology Tools for ArcGIS (Jake Wall. *ArcMET*, Version 10.3.1, 2019; http://www.movementecology.net/arcmet_software.html; accessed on 15 December 2020) [38]. Then, the spatial analysis function of Ethographer in Igor Pro was used for linear interpolation of paths with a 1 s interval to fill the blanks between the data points [39]. We set the detailed time by comparing and confirming the total trip duration from GPS and dive depth data, and then combined the dive depth data with the interpolated GPS data for every second. Points near the nest before and after the foraging trip were excluded. We thus obtained the start time and end time of each dive, with the coordinates (latitude and longitude) of the start and end points in a straight line. Dive depth was determined as the deepest point of the dive, and the coordinates of that point represented the diving location of that dive for spatial analyses. Dives less than 5 m were excluded, as such dives are few [12,40], and are considered as traveling dives.

In each dive, the deepest dive point was indicated as the foraging dive location and it was used to calculate the kernel density. Areas with 95% and 50% kernel density estimates were calculated, representing the foraging and core foraging areas, respectively. In the 95% and 50% kernel density estimates, overlapping sections between the comparison groups were indicated as a polygon. In addition, the overlapping area was calculated as the proportion of the intersecting area to the total area of the two comparison groups [41]. To analyze differences in dive depths in the overlapping areas between groups, the dive depths at the diving locations within the overlapping polygons were obtained. We further calculated the degree of overlap between the foraging areas using the utilization distribution overlap index (UDOI) [42]. UDOI is a product-based index for the calculation of joint-space use [43].

The bathymetric contour was visualized using the GEBCO 2014 Grid (General Bathymetric Chart of the Oceans; https://www.gebco.net/; accessed on 15 December 2020) to create a bathymetry line every 100 m. The diving location was used for kernel density estimation (KDE) in each group (default cell size 100 m × 100 m and search radius 2 km). We categorized the diving districts to distinguish the Maxwell Bay area from the outer oceans. From the bathymetry map, we determined the diving location in each dive. The ArcMET Path Metrics tool was used to calculate movement distance, maximum distance, and trip duration as trip parameters for each individual. Based on these parameters, individuals of the two species within the same colony and those of the same species between the two colonies were compared.

### 2.3. Blood Sampling and Stable Isotope Analysis

Blood samples were collected from 16 adult Chinstrap penguins on 7 January 2018 and from 15 adult Gentoo penguins on 8 January 2018 at NP, and from 13 Chinstrap and 13 Gentoo penguins on 4 January 2018 at AI. To reduce the stress of the GPS-tracked individuals, we selected other breeding birds in the same colonies at similar breeding schedules with two chicks at the nests. The blood sample allows us to estimate the diet niche of the breeding period days to weeks prior to the sample [6,44]. Blood was drawn from the foot vein of breeding penguins at the nest, using a 1 mL sterile syringe. The collected samples were immediately transferred to sterilized e-tubes in the field, and then dried overnight in a drying oven. The dried blood samples were homogenized with a mortar and pestle. Samples were ground into a fine powder and sealed in a tin capsule [45]. Carbon (δ^13^C) and nitrogen (δ^15^N) stable isotopes were analyzed using elemental analyzer isotope ratio mass spectrometry (EA-IRMS, Pyrogenic EA, Vario PYRO CUBE, isotope IRMS, Elementar, UK). Isotopic niche parameters were obtained using the ‘Stable Isotope Bayesian Ellipses in R (SIBER)’ package in R software version 3.6.3 (The R Foundation for Statistical Computing) [46]. Standard ellipses were indicated on the isotopic space of δ^13^C and δ^15^N. Inter- and intra-specific variations in niche width and proportion of overlap were determined from the standard ellipse areas (SEAs) [47]. Probability density distribution was estimated using the Bayesian model at 50%, 75%, and 95% credibility intervals.

### 2.4. Statistics

Statistics were analyzed in R software version 3.6.3 (The R Foundation for Statistical Computing). To compare the inter-specific and intra-specific dive parameters (mean dive duration and dive depth), we used a generalized linear mixed model determined by likelihood ratio tests in R Package ‘lme4′ [48]. For repeated measured dive parameters, we used generalized linear mixed models (GLMMs). Dive duration or dive depth was included as an explanatory variable with repeated measure and the individual ID was included as a random effect in each model. To compare the inter-specific and intra-specific trip parameters (trip duration, maximum distance, and movement distance), we used generalized linear models (GLMs) with gamma error distribution determined by Wald tests in R Package ‘stats’ [49]. Trip duration, maximum distance, or movement distance were included as explanatory variables, and bird species or colony ID were included as response variables. To compare the dive locations, which were categorized into ‘in bay’ or ‘out of bay’ (proportional data), we used GLMs with quasi-binomial distribution determined by Wald tests in the same package. Dive locations (in bay or out of bay) were included as explanatory variables and bird species or colony ID were included as response variables in the models. In our statistical tests, we noted statistical significance when *p*-values were less than 0.05.

### 2.5. Ethical Approval

All research was approved by the Korean Ministry of Foreign Affairs (certificate paper number: ILAD-3146 [14 November 2017]) and according to the current laws of the Republic of Korea (‘Act on Antarctic Activities and Protection of Antarctic Environment’).

## 3. Results

**Figure 1:**
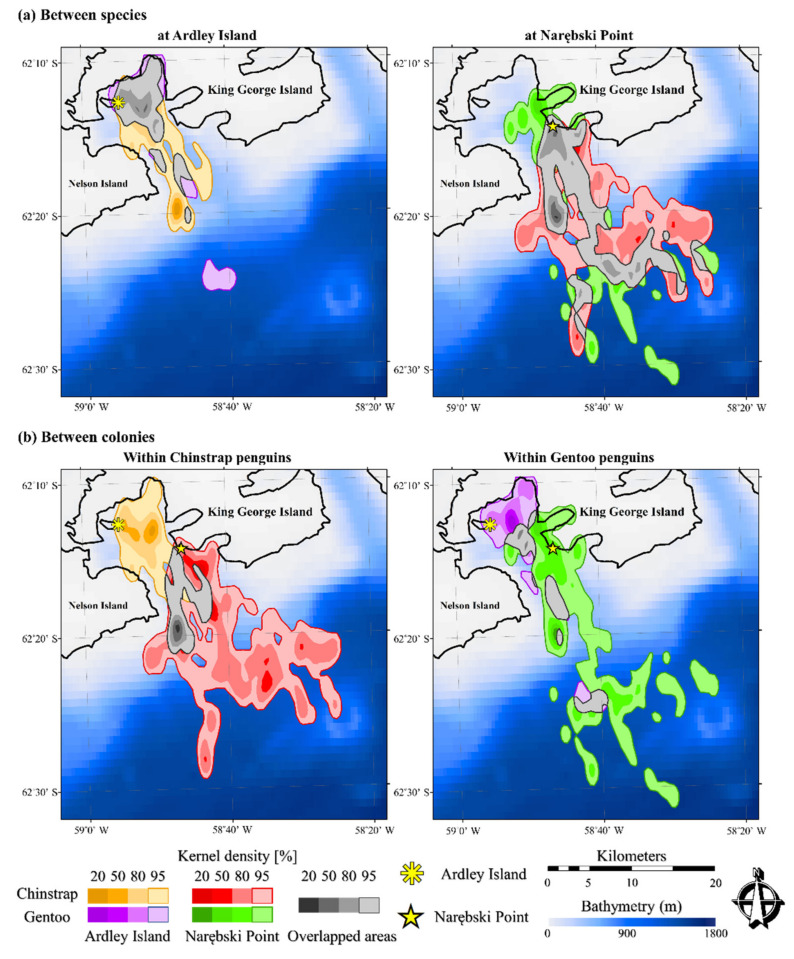
Foraging areas compared between species (Chinstrap vs. Gentoo penguins) (**a**) and between colonies (Ardley Island vs. Narębski Point) (**b**). An asterisk indicates the colony location at Ardley Island and a star indicates the colony at Narębski Point. The foraging areas and the overlapping areas are visualized by kernel density estimation (KDE) values, at 20%, 50%, 80%, and 95%.

A total of 47 foraging trips were obtained from the GPS and diving depth data. Foraging locations of Chinstrap and Gentoo penguins at the two colonies, mapped using KDE values, and the overlapped areas are visualized in Figure 1. The foraging area with 95% kernel density, the core foraging area with 50% kernel density, and the UDOI values are provided in Table 1. In both species, NP penguins exhibited greater foraging areas than AI penguins at 95% and 50% KDE.

Inter-specifically, the overlapping surface intersection area (95% KDE) revealed a large overlap in the foraging areas (47.4% of Chinstrap and 68.1% of Gentoo at AI; 43.4% of Chinstrap and 57.1% of Gentoo at NP) and in the core foraging areas (50% KDE) of both colonies (3.3% of Chinstrap and 7.5% of Gentoo at AI; 11.3% of Chinstrap and 44.2% of Gentoo at NP). Intra-specifically, the overlapping areas were lower than the ones in inter-specific interactions in the foraging areas (within Chinstrap, 37.9% and 13.9% at AI and NP, respectively; within Gentoo, 40.3% and 13.5% at AI and NP, respectively) and in the core foraging areas (within Chinstrap, 27.2% and 12.3% at AI and NP, respectively; within Gentoo, 0% at AI and NP). The values are presented in Table 1. The percentages of overlapping areas were higher in the inter-specific comparison than in the intra-specific comparisons, which was also supported by the UDOI values (Table 1). Inter-specifically, the UDOIs were 0.645 and 0.196 at AI and NP, respectively, whereas those for intra-specific (or inter-colony) comparisons were 0.142 and 0.080 for Chinstrap and Gentoo penguins, respectively.

Dive depths and durations showed that Gentoo penguins at both colonies undertook deeper and longer dives than the Chinstrap penguins in the same colony (Table 2). At the species level, the dive durations of Chinstrap and Gentoo penguins were significantly different at both colonies, whereas significant differences in dive depth were detected at NP (GLM, Wald tests, *p*-values provided in Table 2). At the colony level, Gentoo penguins at NP undertook shallower and shorter dives than those at AI (GLM, *p*-values provided in Table 2); however, no differences were detected in the dive characteristics of Chinstrap penguins.

Trip parameters (trip duration, maximum distance, and movement distance) were not significant in inter-specific comparisons at both colonies (GLM, Wald tests, all *p* > 0.10; Table 2). In intra-specific comparisons, however, Chinstrap penguins showed significant differences in maximum distance, with shorter trip distances from the nests at AI (GLM, Wald tests, χ^2^ = 8.3, *p* = 0.004), and significant differences in movement distance, with shorter distances at AI (GLM, Wald tests, χ^2^ = 4.7, *p* = 0.03).

**Figure 2:**
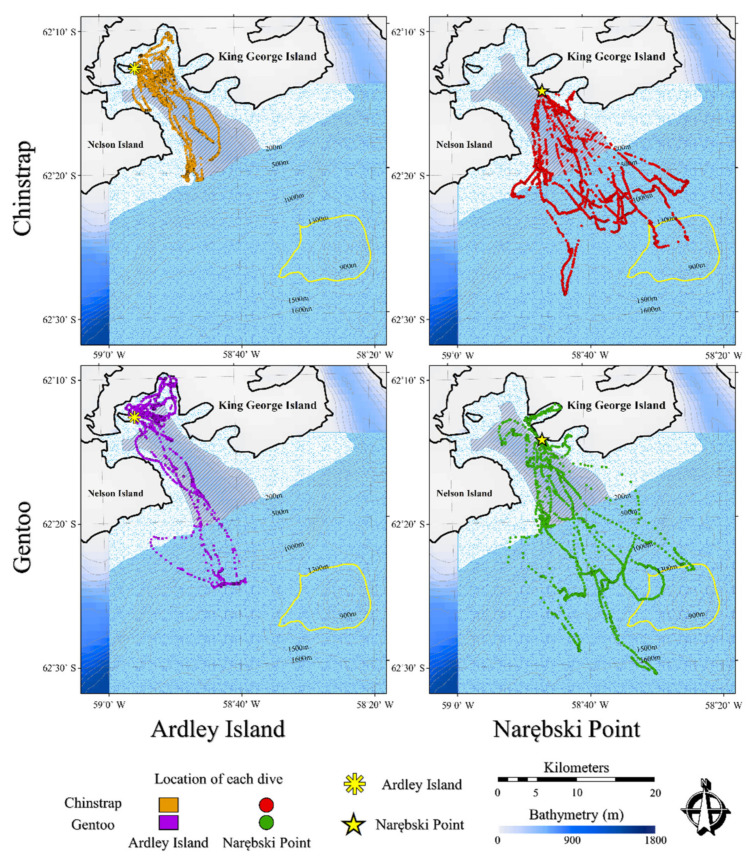
Spatial visualization of the foraging dive locations of Chinstrap and Gentoo penguins at Ardley Island (AI) and Narębski Point (NP). The bathymetric contours are categorized as the On-Shelf area, Maxwell Bay area, and Off-shelf area. In addition, the Orca seamount area is indicated by a yellow contour. Sample sizes were 13 at AI and 19 at NP of Chinstrap and 12 and 20 of Gentoo penguins, respectively.

Dive location did not differ between the two species (GLM, Wald test, χ^2^ = 2.7, *p* = 0.10 at AI; 0.45 at NP, respectively). Intra-specific comparisons indicated that NP penguins moved farther out of the bay to off-shelf areas at both colonies (GLM, *p* = 0.01 for Chinstrap and 0.06 for Gentoo; Figure 2). No AI penguins reached the Orca Seamount area, whereas one NP Chinstrap and two NP Gentoo penguins went to this area.

**Figure 3:**
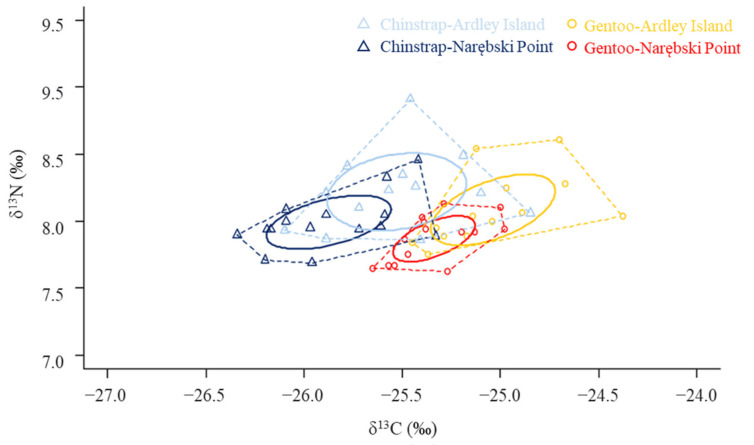
Stable isotope values of δ^13^C and δ^15^N in Chinstrap and Gentoo penguins at Ardley Island and Narębski Point. Standard ellipses indicate 40% prediction. Individual numbers were 13 at AI and 16 at NP of Chinstrap and 13 and 13 of Gentoo penguins, respectively.

Both Chinstrap and Gentoo penguins at AI exhibited higher δ^13^C and δ^15^N values than those at NP (GLM, likelihood ratio test; χ^2^ = 9.8, *p* = 0.002 in δ^13^C; χ^2^ = 12.0, *p* < 0.001 in δ^15^N). In addition, at both colonies, Gentoo penguins had higher δ^13^C than Chinstrap penguins (GLM, likelihood ratio test; χ^2^ = 32.7, *p* < 0.001 in δ^13^C; χ^2^ = 3.2, *p* = 0.07 in δ^15^N). Chinstrap penguins had higher SEA values than Gentoo penguins (Figure 3). The SEAs of Chinstrap penguins were 0.308 at AI and 0.170 at NP, and the values of Gentoo penguins were 0.218 and 0.89, respectively. The SEAs did not indicate any overlap between the two penguin species at NP and a 0.025 overlap was noted at AI. Intra-specific comparisons presented higher overlapping areas (0.154 for Gentoo penguins and 0.159 for Chinstrap penguins; Figure 3).

## 4. Discussion

We revealed the foraging areas of Chinstrap and Gentoo penguins from two neighboring colonies. Utilization distribution (UD) values demonstrated overlapping area use by the two species (43.4–68.1% in 95% UD and 3.3–44.2% in 50% UD), and intra-specific overlaps at the two colonies were lower than inter-specific overlaps (13.5–40.3% in 95% UD and 0–27.2% in 50% UD). UDOI values also indicated that there was a greater overlap of foraging areas between species (0.645 at AI and 0.196 at NP) than within species at the two colonies (0.142 for Chinstrap and 0.80 for Gentoo penguins). These results suggest that foraging areas of the same species from different colonies are highly segregated, supporting our prediction that intra-specific competition could be more intense than inter-specific competition [17,21].

Gentoo penguins are benthic divers with the ability to undertake deep dives [50,51], whereas Chinstrap penguins are epipelagic divers [12,40]. Previous studies at the AI and NP colonies have reported distinct foraging niches of the Gentoo and Chinstrap penguins by differentiating spatial use and diet compositions [12,41], or avoiding foraging time overlaps [30]. Such inter-specific segregation minimizes competition for the same prey sources. However, the extent of inter-specific niche segregation was not compared with intra-specific competition at the neighboring colonies. Taking intra-specific comparisons into consideration, we concluded that inter-colony partitioning occurred, leading to reduced niche overlap, and it appeared to be greater than inter-specific segregation.

In intra-specific competition, differences in the colony sizes would be the driving force for segregation. A recent study showed that Adélie penguins (*Pygoscelis adeliae*) at two neighboring colonies segregated the foraging areas according to the colony size [19]. In the present study, Chinstrap penguins at AI were remarkably outnumbered by those at NP (20 vs. 2918 pairs), whereas Gentoo penguins at AI outnumbered those at NP (7227 vs. 2604 pairs). The difference in colony size appeared to be positively correlated to the foraging area of Chinstrap penguins. The 95% UD indicated that Chinstrap penguins at AI were restricted to a 97.7 km^2^ area in the bay, whereas those at NP utilized an area of 266.5 km^2^, diving far south in the open water. Larger seabird colonies exhibit broader foraging ranges [52], which can restrict the foraging ranges of smaller colonies. However, the results for Gentoo penguins were not consistent with the colony size explanation. Considering the higher Gentoo numbers at AI, the foraging area was predicted to be larger for the AI colony. However, the 95% UD area for AI (67.7 km^2^) was smaller than that for NP (202.5 km^2^). We speculate that Gentoo penguins at the two colonies employ different foraging strategies. In the case of the dive characteristics, only Gentoo penguins exhibited a significant difference in dive depth and duration, i.e., Gentoo penguins at NP performed deeper and longer dives than those at AI. Although intra-specific niche partitioning is not commonly observed, we attribute such a partitioning to the flexible foraging behaviors of Gentoo penguins. These penguins employ highly variable strategies, depending on the individual [53], local prey availability [54], and year [55]. As they are generalist foragers, such variability may lead to vertically different foraging behaviors between the two colonies.

The results of stable isotope values demonstrated both inter- and intra-specific competition, exhibiting different foraging habitats (δ^3^C) and trophic levels (δ^15^N) between the species and colonies. This suggests that the two penguin species and the two colonies occupy different isotopic niches. The higher δ^13^C values of Gentoo penguins indicate that Gentoo penguins could have more inshore prey habits than Chinstrap [33,56]. In addition, the higher δ^15^N of AI penguins suggest that they could forage higher trophic preys than NP penguins. This result is in accordance with previous studies that Gentoo penguins were more benthic divers than Chinstrap penguins at NP and that Chinstrap penguins performed frequent epipelagic dives in this area [12,30,31]. Although Chinstrap and Gentoo penguins share a common main prey species, i.e., the Antarctic krill, they could separate their ecological niches by targeting different types of krill in different marine habitats. A recent study demonstrated high variations in the stable isotope values of Antarctic krill, with mean isotopic values varying by as much as 2.4‰ in δ^15^N, and the δ^15^N and δ^13^C values were positively correlated with krill length [57]. This indicates that Chinstrap and Gentoo penguins, both of which predate on krill, may also have variations in their isotopic values, which may be attributed to differing prey size composition or oceanographic factors. Despite the shorter trip hours and distances of AI penguins, the SEAs values, which imply the isotopic niche width [47,58], exhibited that they had higher widths. This implies that the AI penguins could focus on foraging more diverse prey sources in the bay area.

Comparing the isotopic niches between and within species, it showed that there were lower inter-specific overlaps than intra-specific overlaps. This suggests that, despite the low intra-specific spatial overlaps, diets of conspecifics from different colonies remained more similar, resulting in the higher isotopic niche overlaps. The spatial segregation between species sharing similar diets supports our previous prediction that intra-specific competition is stronger than inter-specific competition.

Because our study sites were in the bay area, the differences between the two breeding locations could result in spatial segregation between conspecific individuals. NP birds are located in the deeper area, approximately 3 km further from the open sea out of the bay, so they may have more energy expenditures than AI birds. Thus, foraging spatial segregation between conspecifics may be enforced by central place constraints such as limited availability and traveling distance [25,26,27].

In addition to the foraging space use, differences in the breeding schedules of the two species should be taken into consideration for inter- and intra-specific comparisons. The present study was conducted in December and January; however, slight differences occur in the breeding time of the two species. Subsampling from 500 nests showed that the mean hatching date of Gentoo penguin chicks was estimated on 17 December and the mean hatching date of Chinstrap penguin chicks was on 23 December [35,36]. This six-day gap between the hatching dates of the two species may reduce the overlap of the breeding period, to cater for the greater need for food to feed the chicks. This is in accordance with a previous study on Adélie, Gentoo, and Chinstrap penguins, which reported that the three species might reduce niche overlap by varying their breeding times [4]. Thus, the two species at our study sites may also have adopted a similar strategy to minimize the foraging peak times by differentiating the start of breeding. This may enable the two sympatric species to share a large overlapping foraging space, while preventing the same species at the two neighboring colonies from occupying a foraging niche due to their similar breeding schedules.

## 5. Conclusions

We demonstrated the foraging space use of Chinstrap and Gentoo penguins from two neighboring colonies, inferred from GPS and dive depth data, along with inter-specific and intra-specific comparisons. Although stable isotope analysis indicated that the diets of conspecifics from different colonies remained more similar than those of allospecifics, comparison of the overlapping areas revealed that intra-specific segregation was higher than inter-specific partitioning. We suggest that high intra-specific competition may lead to spatial segregations between colonies.

In future studies, it will be interesting to monitor their foraging behaviors through a long-term study. Our study area has undergone rapid changes in its prey environments since the 1980s, with climate change in the Antarctic Peninsula and the decrease of δ^13^C and δ^15^N values reported in Adélie, Chinstrap, and Gentoo penguins [44]. Gentoo penguins are known as generalists and Chinstrap penguins as specialists, based on their foraging strategies [18]. Thus, inter-annual comparisons would reveal how generalists and specialists respond to environmental changes, related to their ecological niche segregations.

## Figures and Tables

**Table 1:** 95% and 50% kernel density estimation (KDE) area (in km^2^), overlapping surface intersection area (%), and utilization distribution overlap index (UDOI) values for inter- and intra-specific comparisons of sympatrically breeding Chinstrap and Gentoo penguins at Ardley Island (AI) and Narębski point (NP).

	Chinstrap		Gentoo		Inter-Specific Comparison		Intra-Specific Comparison	
					at AI	at NP	within Chinstrap	within Gentoo
	AI (*n* = 13)	NP (*n* = 19)	AI (*n* = 12)	NP (*n* = 20)	Chinstrap/Gentoo (% Overlapped)	Chinstrap/Gentoo (% Overlapped)	AI/NP (% Overlapped)	AI/NP (% Overlapped)
95% KDE	97.7	266.5	67.7	202.5	47.4/68.1	43.4/57.1	37.9/13.9	40.3/13.5
50% KDE	9.2	20.3	4	5.25	3.3/7.5	11.3/44.2	27.2/12.3	0/0
UDOI					0.645	0.196	0.142	0.080

**Table 2:** Inter-specific and intra-specific comparisons for dive parameters (mean dive duration and dive depth) with generalized linear mixed models (GLMMs) and for trip parameters (trip duration, maximum distance, and movement distance) and dive location (in bay or out of bay) with generalized linear models (GLMs). * indicates significant differences (*p* < 0.05).

	Chinstrap (Mean ± SD)		Gentoo (Mean ± SD)		Inter-Specific Comparison		Intra-Specific Comparison	
					At AI	At NP	For Chinstrap	For Gentoo
	AI (*n* = 13)	NP (*n* = 19)	AI (*n* = 12)	NP (*n* = 20)	*p*-Value	*p*-Value	*p*-Value	*p*-Value
*Dive parameter*								
Mean dive duration (s)	67.3 ± 15.6	75.9 ± 15.6	85.8 ± 16.6	107.6 ± 18.5	0.01 *	<0.001 *	0.25	0.01 *
Dive depth (m)	27.7 ± 12.6	33.1 ± 12.3	32.1 ± 12.4	45.7 ± 15.1	0.21	0.04 *	0.40	0.01 *
*Trip parameter*								
Trip duration (h)	6.6 ± 2.8	8.8 ± 3.2	7.8 ± 3.1	8.7 ± 3.5	0.33	0.98	0.08	0.49
Maximum distance (km)	8.7 ± 5.4	16.6 ± 7.7	10.7 ± 9.4	16.6 ± 10.8	0.45	0.99	0.004 *	0.17
Movement distance (km)	28.5 ± 16	44.1 ± 18.1	33.0 ± 21.2	47.1 ± 23.6	0.56	0.72	0.03 *	0.14
*Dive location*								
In bay (on-shelf area and Maxwell Bay)	99.2 ± 2.77	52.73 ± 33.29	89.32 ± 19.06	63.92 ± 38.02	0.10	0.45	0.01 *	0.06
Out of bay (off-shelf area)	0.8 ± 2.77	47.27 ± 33.29	10.68 ± 19.06	36.08 ± 38.02				

## Data Availability

The GPS and depth data was deposited in the Korea Polar Research Institute data repository (https://dx.doi.org/doi:10.22663/KOPRI-KPDC-00001615.1).

## Supplementary Materials

The following are available online at https://www.mdpi.com/2076-2615/11/2/482/s1, Table S1: The number of GPS (GPL400 and F3G) and time-depth (M190 and ORI400) recorders that we used in each colony on Chinstrap and Gentoo penguins.
