# Supplementary material for: Inter-Specific and Intra-Specific Competition of Two Sympatrically Breeding Seabirds, Chinstrap and Gentoo Penguins, at Two Neighboring Colonies
Source: Animals (Basel). 2021 Feb 11;11(2):482. doi: 10.3390/ani11020482 (PMC7918894; doi:10.3390/ani11020482)
Supplement: Supplementary file 1 [file animals-11-00482-s001.pdf]

Supplemental Material Table S1

Table S1. The number of GPS (GPL400 and F3G) and time-depth recorders (M190 and ORI400) that we used in each colony on Chinstrap and Gentoo penguins.

	Ardley Island		Narębski point	
	Chinstrap	Gentoo	Chinstrap	Gentoo
GPL400	8	8	3	5
F3G and M190	5	-	8	11
F3G and ORI400	-	4	8	4
